# Ciliary localization of GPR75 promotes fat accumulation in mice

**DOI:** 10.1172/JCI185059

**Published:** 2024-10-01

**Authors:** Marcelo Chávez, Anushweta Asthana, Peter K. Jackson

**Affiliations:** Baxter Laboratory for Stem Cell Biology, Department of Microbiology and Immunology, Stanford University School of Medicine, Stanford, California, USA.

## Abstract

Obesity is a growing public health concern that affects the longevity and lifestyle of all human populations including children and older individuals. Diverse factors drive obesity, making it challenging to understand and treat. While recent studies highlight the importance of GPCR signaling for metabolism and fat accumulation, we lack a molecular description of how obesogenic signals accumulate and propagate in cells, tissues, and organs. In this issue of the *JCI*, Jiang et al. utilized germline mutagenesis to generate a missense variant of GRP75, encoded by the *Thinner* allele, which resulted in mice with a lean phenotype. GPR75 accumulated in the cilia of hypothalamic neurons. However, mice with the *Thinner* allele showed defective ciliary localization with resistance to fat accumulation. Additionally, GPR75 regulation of fat accumulation appeared independent of leptin and ADCY3 signaling. These findings shed light on the role of GPR75 in fat accumulation and highlight the need to identify relevant ligands.

## Obesity is regulated by GPCR signaling

It is estimated that 74% of adults in the United States are either overweight or obese, and over 10% have type 2 diabetes (T2D). Worldwide, T2D accounts for nearly 90% of the approximately 537 million individuals with diabetes. The number of those affected with T2D is growing at alarming rates in children and young adults (1, 2). Obese individuals have a higher risk of heart disease, stroke, sleep apnea, metabolic dysfunction–associated steatotic liver disease (MASLD), cancer, kidney failure, and premature death (1, 3). Obesity is a complex polygenic disease, but with no singular, prevalent obesity genes. Nonetheless, several consistent signaling pathways regulate feeding, satiety, and adipogenesis and control fat accumulation. Many involve signaling via GPCRs that control neuroendocrine processes. Mutations in several GPCRs, and notably those in the stimulatory G protein α subunit (Gαs) and specific adenylate cyclases, including ADCY3, are strongly linked to severe obesity (4–6). Many of these GPCRs respond to neuroendocrine peptides released in the feeding response to enable metabolic responses such as secretion of insulin or other neuropeptides. These factors can traffic via serum to the brain to activate neuronal receptors that suppress feeding. Particularly, in response to feeding, glucagon-like peptide 1 (GLP-1) is released by the intestinal lining and in turn accelerates insulin secretion and reduces glucagon secretion in pancreatic islets. GLP-1 also slows gastric emptying and regulates food intake. Notably, GLP1 receptor agonists are effective treatments for obesity and diabetes (1).

## Primary cilia signaling defects in monogenic obesity syndromes

Primary cilia are small, solitary, hair-like structures that project apically from many cells. They constitute cellular signaling hubs where multiple transduction pathways converge (7). An increasing number of GPCRs require ciliary localization for proper function (8–11). These ciliary GPCRs are ferried into primary cilia via the interaction with the TUBBY/TULP3 protein family TULP3), known to bind to phosphoinositides and an ancient ciliary protein–trafficking complex, IFT-A (12, 13).

Primary cilia dysfunction causes ciliopathies, exemplified by the Bardet-Biedl syndrome, which is characterized by obesity, retinal degeneration, anosmia, and various morphogen defects (14). Earlier studies sought to identify important ciliary GPCRs expressed in the hypothalamus, the key feeding center of the brain (9, 15). These studies identified eight ciliary GPCRs expressed in the brain, including members of the neuropeptide Y family. Notably, the NPY2R receptor failed to localize to cilia in the mouse hypothalamus and failed to respond to the anorexigenic ligand PYY3-36 (9). Human mutations that affect the ciliary localization of the melanocortin receptor MC4R and reduce adenylyl cyclase signaling at the primary cilia of hypothalamic neurons are associated with increased body weight and cause obesity in genetic mouse models (15, 16).

## Mutagenesis screening reveals the GPR75 controller of feeding

**Figure 1:**
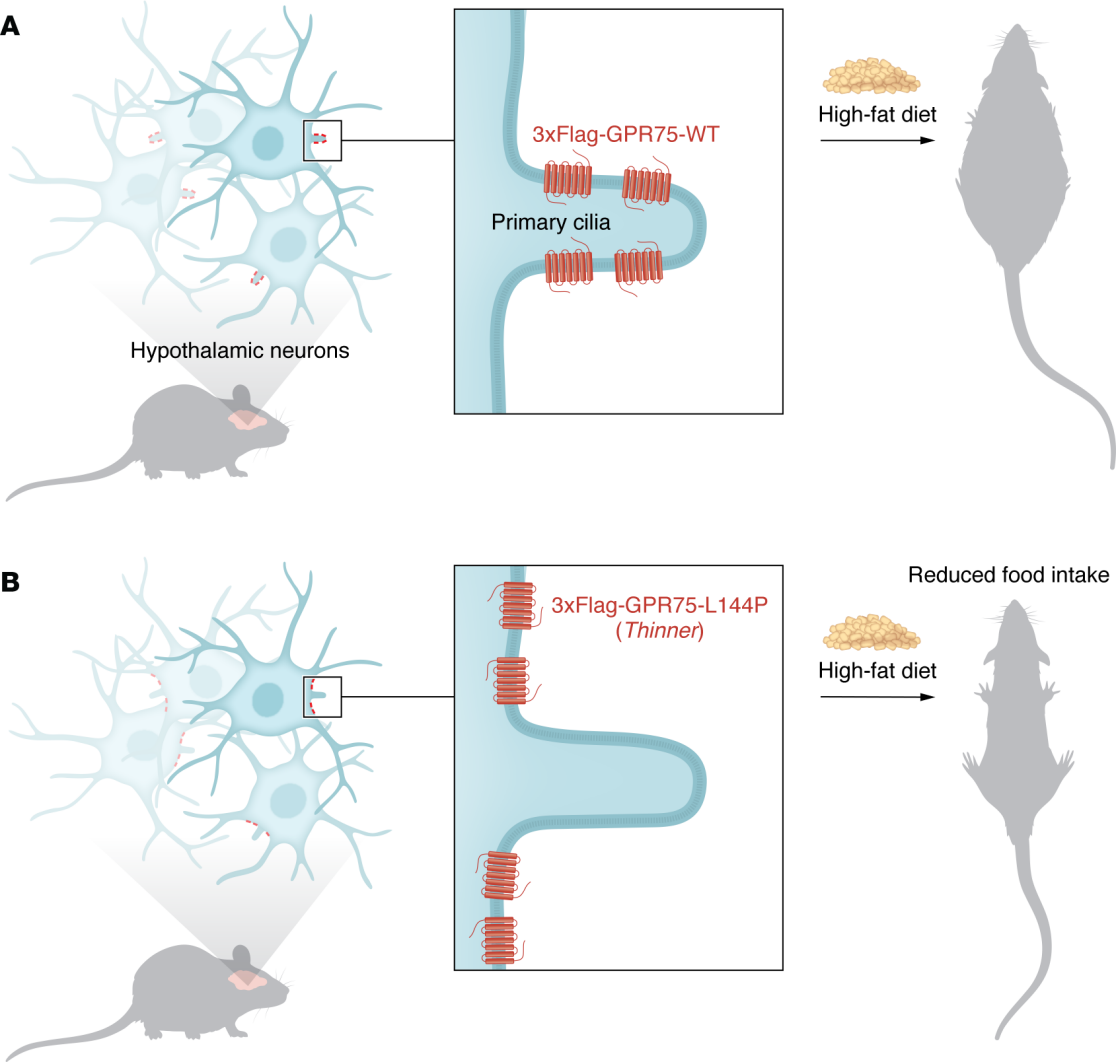
Mice with the *Gpr75* allele *Thinner* are resistant to weight gain when exposed to a HFD. (**A**) 3xFlag-GPR75 is expressed in primary cilia of hypothalamic neurons, and wild-type mice become obese when fed a HFD. (**B**) The 3xFlag-GPR75-L144P mutant fails to localize to primary cilia, and mice carrying this mutation show resistance to weight gain.

In this issue of the *JCI*, Jiang et al. (17) identified a missense allele of Gpr75, *Thinner*, through *N*-ethyl-*N*-nitrosourea (ENU) germline mutagenesis. *Thinner* showed a lean phenotype in C57BL/6J mice, with reduced body weight and fat mass (Figure 1). The mutation was mapped and sequenced, revealing a missense allele at lysine 144 (L144P). This mutation did not affect overall GPR75 protein levels. The *GPR75/Gpr75* locus had previously been identified in a large exome-sequencing screen of patients, showing patients with notably reduced body mass, and in knockout mice with decreased food intake and reduced fat deposits in the liver (18–21).

## *Gpr75^–/–^* mice show decreased food intake

Jiang and authors further examined the physiological mechanism behind reduced body mass (17). The high-fat diet (HFD) is a useful model for accelerating caloric intake and enhanced weight gain. The authors constructed a null allele of *Gpr75* and found that, relative to WT mice, *Gpr75^–/–^* mice were resistant to fat accumulation when challenged with a HFD. Notably, throughout the 8-week HFD feeding experiment, lean mass remained unchanged between the *Gpr75^–/–^* and wild-type mice, while differences in fat mass were evident within two weeks of HFD feeding. The authors further demonstrated that on a HFD, *Gpr75*^–/–-^ mice had smaller adipocytes and reduced ectopic adipogenesis in brown fat and liver (17).

Examination of feeding behavior confirmed that reduced feeding was strongly linked to leanness. A pair-feeding control experiment rescued the differences in weight gain, supporting the hypothesis of altered feeding control. Using metabolic cages to analyze other potential contributions to the lean phenotype, Jiang and authors found no changes in energy expenditure, respiration, or physical activity. They also noted no change in serum metabolites except for reduced leptin, which is an expected reflection of reduced fat mass.

## Ciliary GPR75 does not respond to known ligands

To better understand the nature of GPR75 signaling, Jiang and colleagues generated a 3xFlag-tagged *Gpr75* mouse knockin model, which showed that endogenous GPR75 was mostly expressed in the CNS (17). Notably, GPR75 dramatically localized to neuronal primary cilia. Essentially all neurons in the brain are ciliated, and the current thinking is that neuronal cilia respond to neuroendocrine circuits in the brain to regulate synaptic neural signaling by both acute and long-term mechanisms (22). The presence of GPR75 in the brain suggests that peripheral feeding signals, possibly transported to the cerebrospinal fluid (CSF), would contain key ligands for regulating feeding. Cultured hypothalamic neurons from the *Gpr75*-knockin mice also revealed that GPR75 localized to primary cilia. Notably, the missense allele did not affect GPR75 expression but failed to localize to cilia (Figure 1) (17).

Previous publications suggest that the Rantes factor CCL5 and the eicosanoid 20-HETE may serve as GPR75 ligands (23, 24). Jiang et al. did not detect ligand activity for these factors, suggesting that the correct ligand remains unknown (17).

## *Gpr75* mutants do not rescue specific obesity mutations

Jiang and authors additionally examined feeding regulation by GPR75 in the context of two known modulators of metabolism (17). Functional deficiencies in leptin and ADCY3 disrupt feeding control and promote obesity and T2D (6, 25). Through ENU screening, the authors identified an *Adcy3*-mutant mouse (*Adcy3^L278H/L278H^*) that develops obesity. To examine coupling of GPR75 to known anorexogenic signaling, the authors crossed *Gpr75^–/–^* mice with *Lep^ob/ob^* mice and *Adcy3^L278H/L278H^* mice. Interestingly, *Gpr75* knockout failed to rescue the obesity phenotype in both crosses. These results suggest that signaling of both leptin and ADCY3 , which typically suppresses feeding control, work more generally to drive obesity and that GPR75 signaling is either epistatic or phenotypically less powerful than these obesity circuits.

## Concluding remarks and perspectives

Jiang et al. establish that ciliary localization of GPR75 is key for its activity and ability to regulate fat tissue accumulation (17). However, these findings open several questions. Which neuronal cell populations are responsible for the GPR75 fat-accumulating phenotype? Better identification of specific brain centers will provide additional clues for the nature of the signal sensed by GPR75. Does GPR75 detect any component of a HFD? There may be olfactory- or feeding-related components of a HFD, notably lipids, that signal to enhance feeding. Either directly or indirectly, these could affect factors collected in CSF and activate GPR75 to positively stimulate directed feeding for specific lipid-rich foods.

Another open question pertains to the basis of GPR75 variant mislocalization. Can only signaling-competent molecules of GPR75 localize to the cilium? The GPR75 L144P mutation occurs within helix TM3; it may be that this substitution could destabilize the helix and alter the position of nearby residues to interfere with binding partners required for trafficking or signaling. More experiments are needed to deconvolve the molecular details of GPR75 signaling.

The current study poses the question: Is the GPR75 ligand present in high-fat food as an exogenous factor with sensory value, or does GPR75 work indirectly to mediate signals that are part of a known feeding anorexigenic signaling pathway? Future directions should include identification of those factors linked to the HFD and, given the neural localization of the receptor, determine whether those factors are present in CSF.

## Version 1 10/01/2024

Electronic publication
